# Reduced Animal Models Fitting Only Equations for Phenotyped Animals

**DOI:** 10.3389/fgene.2021.637626

**Published:** 2021-03-22

**Authors:** Mohammad Ali Nilforooshan, Dorian Garrick

**Affiliations:** ^1^Livestock Improvement Corporation, Hamilton, New Zealand; ^2^AL Rae Centre for Genetics and Breeding, School of Agriculture, Massey University, Palmerston North, New Zealand

**Keywords:** animal model, phenotyped, reduced model, back-solve, relationship matrix

## Abstract

Reduced models are equivalent models to the full model that enable reduction in the computational demand for solving the problem, here, mixed model equations for estimating breeding values of selection candidates. Since phenotyped animals provide data to the model, the aim of this study was to reduce animal models to those equations corresponding to phenotyped animals. Non-phenotyped ancestral animals have normally been included in analyses as they facilitate formation of the inverse numerator relationship matrix. However, a reduced model can exclude those animals and obtain identical solutions for the breeding values of the animals of interest. Solutions corresponding to non-phenotyped animals can be back-solved from the solutions of phenotyped animals and specific blocks of the inverted relationship matrix. This idea was extended to other forms of animal model and the results from each reduced model (and back-solving) were identical to the results from the corresponding full model. Previous studies have been mainly focused on reduced animal models that absorb equations corresponding to non-parents and solve equations only for parents of phenotyped animals. These two types of reduced animal model can be combined to formulate only equations corresponding to phenotyped parents of phenotyped progeny.

## 1. Introduction

Computational limitations have been a major challenge facing animal breeders, especially for solving large mixed model equations, such as those including multiple traits and millions of animals. Computational speed, power and technology have advanced, making most of the computations that were impossible in the past, feasible. However, the amount of data is growing rapidly, and the complexity of evaluation models are increasing. Henderson (1974) introduced the concepts of equivalent models and reduced models to animal breeding. Any linear model can be written in various forms of equivalent models yielding the same first and second moments of the data (Henderson, 1985). A reduced model is an equivalent model to the full model, allowing computational simplifications by reduction in the number of equations to be solved.

Limited by available memory to store the entire set of equations, Henderson (1974) proposed an equivalent reduced model in which the equations for fixed herd effects were absorbed into the equations for sire effects. The full model explicitly fitted herd and sire effects. Generally, absorption is a model rank reduction (reduced number of equations to be solved), by which some effects get absorbed into other effects with no influence on the solutions of the remaining effects in the model.

Reduced models that absorbed multiple-trait equations for random effects were first introduced to animal models by Quaas and Pollak (1980), where they showed that the best linear unbiased predictions (BLUP) of parents (with phenotyped progeny) can be obtained from a reduced model that only includes equations for breeding values for parents. This was achieved by absorbing equations of non-parent individuals (and parents with no phenotyped progeny). The BLUP of breeding values for non-parent individuals can then be back-solved given the BLUP of breeding values for their parents. The concept of reduced models was extended to animal models incorporating quantitative trait loci, introduced by Fernando and Grossman (1989), and was also based on absorbing equations corresponding to non-parent individuals (Cantet and Smith, 1991; Hoeschele, 1993; Arendonk et al., 1994; Saito and Iwaisaki, 1996, 1997).

Henderson (1986) proposed the estimation of variance components in reduced (based on the methodology of Quaas and Pollak, 1980) rather than full animal models for single traits and single records, based on minimum variance quadratic unbiased estimation (MIVQUE), restricted maximum likelihood (REML) by iterated MIVQUE, and REML by the expectation-maximization (EM, Dempster et al., 1977) algorithm. Providing numerical examples, he showed that the reduced model can be used for estimation of variances, converging to the same solutions obtained by the full model.

This study revisited the reduced animal model from a different perspective by only including equations corresponding to phenotyped animals in the model. In a full model, non-phenotyped parents do not contribute any phenotypic information to the model, but make it easier to account for similarity between animals with phenotypes when they are included in the construction of the inverse numerator relationship matrix. This form of reduced animal model that absorbs the effects of non-phenotyped breeding values is first explained in the context of a simple univariate animal model, and then extended to several other forms of animal model. Numerical examples and R scripts are provided to facilitate understanding.

## 2. Methods

Animal models can be expressed and fitted in various forms and combinations. In this section, some equivalent forms of animal model, their size reduction (absorption) to phenotyped animals (denoted as *p*), and approach to back-solve solutions corresponding to non-phenotyped animals (denoted as *n*) are presented.

### 2.1. Basic Animal Model

In its simplest commonly-used form (**y** = **Xb** + **Zu** + **e**), an animal model contains the vectors of phenotypes (**y**), fixed effects (**b**), random direct additive genetic effects [$\text{u}\sim N\left(0,\text{A}\sigma_{u}^{2}\right)$, representing breeding values] and random residuals $\left(\text{e}\sim N\left(0,\text{I}\sigma_{e}^{2}\right)\right)$, with $\sigma_{u}^{2}$ and $\sigma_{e}^{2}$ being the corresponding known variances, and **A** being the numerator relationship matrix. Matrices **X** and **Z** relate phenotypes to fixed effects and animals, respectively. In matrix notation solutions to this single-trait model are obtained by solving the equation system:

(1) $$\begin{array}{l} \left[\begin{array}{cc} {\text{X}}^{\prime }\text{X} & {\text{X}}^{\prime }\text{Z}\\ {\text{Z}}^{\prime }\text{X} & {\text{Z}}^{\prime }\text{Z}+\lambda {\text{A}}^{-1} \end{array}\right]\left[\begin{array}{c} \hat{\text{b}}\\ \hat{\text{u}} \end{array}\right]=\left[\begin{array}{c} {\text{X}}^{\prime }\text{y}\\ {\text{Z}}^{\prime }\text{y} \end{array}\right], \end{array}$$

where $\hat{\text{b}}$ is a vector of solutions for fixed effects (**b**) and $\hat{\text{u}}$ is the vector of best linear unbiased predictions for **u** (known as estimated breeding values) and $\lambda =\sigma_{e}^{2}/\sigma_{u}^{2}$. Permuting rows and columns of the sparse matrix **A**^−1^ so that non-phenotyped animals appear before phenotyped animals, ${\text{A}}^{-1}=\left[\begin{array}{cc} {\text{A}}^{nn} & {\text{A}}^{np}\\ {\text{A}}^{pn} & {\text{A}}^{pp} \end{array}\right]$, ${\hat{\text{u}}}^{\prime }=\left[\begin{array}{cc} {\hat{\text{u}}}_{n}^{\prime } & {\hat{\text{u}}}_{p}^{\prime } \end{array}\right]$and **Z** = $\text{Z}=\left[\begin{array}{cc} 0 & {\text{Z}}_{p} \end{array}\right]$, where **0** is a matrix with elements equal to zero. Here, **0** has number of rows equal to the number of phenotypes and number of columns equal to the number of non-phenotyped animals. Then, Equation (1) can be written as:

(2) $$\begin{array}{l} \left[\begin{array}{ccc} {\text{X}}^{\prime }\text{X} & 0 & {{\text{X}}^{\prime }\text{Z}}_{p}\\ 0 & \lambda {\text{A}}^{nn} & \lambda {\text{A}}^{np}\\ {\text{Z}}_{p}^{\prime }\text{X} & \lambda {\text{A}}^{pn} & {\text{Z}}_{p}^{\prime }{\text{Z}}_{p}+\lambda {\text{A}}^{pp} \end{array}\right]\left[\begin{array}{c} \hat{\text{b}}\\ {\hat{\text{u}}}_{n}\\ {\hat{\text{u}}}_{p} \end{array}\right]=\left[\begin{array}{c} {\text{X}}^{\prime }\text{y}\\ 0\\ {\text{Z}}_{p}^{\prime }\text{y} \end{array}\right]. \end{array}$$

Permuting **y** so that animals are in the same order as **A**^*pp*^, **Z**_*p*_ = **I**. Then,

(3) $$\begin{array}{l} \left[\begin{array}{ccc} {\text{X}}^{\prime }\text{X} & 0 & {\text{X}}^{\prime }\\ 0 & \lambda {\text{A}}^{nn} & \lambda {\text{A}}^{np}\\ \text{X} & \lambda {\text{A}}^{pn} & \text{I}+\lambda {\text{A}}^{pp} \end{array}\right]\left[\begin{array}{c} \hat{\text{b}}\\ {\hat{\text{u}}}_{n}\\ {\hat{\text{u}}}_{p} \end{array}\right]=\left[\begin{array}{c} {\text{X}}^{\prime }\text{y}\\ 0\\ \text{y} \end{array}\right]. \end{array}$$

If we absorb the equations corresponding to breeding values for non-phenotyped animals by modifying the equations corresponding to breeding values for phenotyped animals, Equation (3) is transformed to Equation (4), which contains breeding values only for phenotyped animals:

(4) $$\begin{array}{l} \left[\begin{array}{cc} {\text{X}}^{\prime }\text{X} & {\text{X}}^{\prime }\\ \text{X} & \text{I}+\lambda \Phi \end{array}\right]\left[\begin{array}{c} \hat{\text{b}}\\ {\hat{\text{u}}}_{p} \end{array}\right]=\left[\begin{array}{c} {\text{X}}^{\prime }\text{y}\\ \text{y} \end{array}\right], \end{array}$$

where

(5) $$\begin{array}{l} \Phi ={\text{A}}^{pp}-{\text{A}}^{pn}{\left({\text{A}}^{nn}\right)}^{-1}{\text{A}}^{np}. \end{array}$$

Derivation of **Φ** is provided later in this subsection. Considering equations corresponding to breeding values for non-phenotyped animals in Equation (3), we have the identity:

(6) $$\begin{array}{l} {\text{A}}^{nn}{\hat{\text{u}}}_{n}=-{\text{A}}^{np}{\hat{\text{u}}}_{p}. \end{array}$$

Thus, ${\hat{\text{u}}}_{n}$ can be back-solved from **A**^*nn*^, **A**^*np*^, and ${\hat{\text{u}}}_{p}$. Comparing Equations (1) and (4), **y** = **Xb** + **Zu** + **e** is reduced to **y** = **Xb** + **u**_*p*_ + **e**. Thus, phenotypes depend only on the genetic merit of phenotyped animals and are independent from the genetic merit of their non-phenotyped relatives. The reduced model has a different structure of additive genetic variance $\left({\text{u}}_{p}\sim N\left(0,\Phi^{-1}\sigma_{u}^{2}\right)\text{ vs}.\;\text{u}\sim N\left(0,\text{A}\sigma_{u}^{2}\right)\right)$.

Consider the typically dense matrix $\text{A}=\left[\begin{array}{cc} {\text{A}}_{nn} & {\text{A}}_{np}\\ {\text{A}}_{pn} & {\text{A}}_{pp} \end{array}\right].$ The following mixed model equations are equivalent to Equation (1), but can only be used to directly predict $\hat{\text{b}}$ and ${\hat{\text{u}}}_{p}$, and it involves no changes in model assumptions (Henderson, 1986):

(7) $$\begin{array}{l} \left[\begin{array}{cc} {\text{X}}^{\prime }\text{X} & {\text{X}}^{\prime }\\ \text{X} & \text{I}+\lambda {\text{A}}_{pp}^{-1} \end{array}\right]\left[\begin{array}{c} \hat{\text{b}}\\ {\hat{\text{u}}}_{p} \end{array}\right]=\left[\begin{array}{c} {\text{X}}^{\prime }\text{y}\\ \text{y} \end{array}\right]. \end{array}$$

The above representation of the model is useful to prove the derivation of **Φ**, but not computationally efficient, because **A**_*pp*_ is generally not sparse and it cannot be inverted using the indirect inversion methods of Henderson (1976) or Quaas (1976). Equating row 3 of Equation (3) $\left(\text{X}\hat{\text{b}}+\lambda {\text{A}}^{pn}{\hat{\text{u}}}_{n}+\left(\text{I}+\lambda {\text{A}}^{pp}\right){\hat{\text{u}}}_{p}=\text{y}\right)$ and row 2 of Equation (7) $\left(\text{X}\hat{\text{b}}+\left(\text{I}+\lambda {\text{A}}_{pp}^{-1}\right){\hat{\text{u}}}_{p}=\text{y}\right)$ results in $\lambda {\text{A}}^{pn}{\hat{\text{u}}}_{n}+\left(\text{I}+\lambda {\text{A}}^{pp}\right){\hat{\text{u}}}_{p}=\left(\text{I}+\lambda {\text{A}}_{pp}^{-1}\right){\hat{\text{u}}}_{p}$, which is rearranged to ${\text{A}}^{pn}{\hat{\text{u}}}_{n}=\left({\text{A}}_{pp}^{-1}-{\text{A}}^{pp}\right){\hat{\text{u}}}_{p}$. Solving the obtained equation with Equation (6):

$$\begin{array}{l} \;\{\begin{array}{c} {\text{A}}^{pn}{\hat{\text{u}}}_{n}=({\text{A}}_{pp}^{-1}-{\text{A}}^{pp}){\hat{\text{u}}}_{p}\\ {\text{A}}^{nn}{\hat{\text{u}}}_{n}=-{\text{A}}^{np}{\hat{\text{u}}}_{p}\;(\text{Equation }6) \end{array}\\ \Rightarrow \{\begin{array}{c} {\text{A}}^{pn}{\hat{\text{u}}}_{n}=({\text{A}}_{pp}^{-1}-{\text{A}}^{pp}){\hat{\text{u}}}_{p}\\ \;{\hat{\text{u}}}_{n}=-{\left({\text{A}}^{nn}\right)}^{-1}{\text{A}}^{np}{\hat{\text{u}}}_{p} \end{array}\\ \Rightarrow -{\text{A}}^{pn}{\left({\text{A}}^{nn}\right)}^{-1}{\text{A}}^{np}{\hat{\text{u}}}_{p}=({\text{A}}_{pp}^{-1}-{\text{A}}^{pp}){\hat{\text{u}}}_{p}\\ \Rightarrow {\text{A}}^{pn}{\left({\text{A}}^{nn}\right)}^{-1}{\text{A}}^{np}={\text{A}}^{pp}-{\text{A}}_{pp}^{-1}\\ \Rightarrow {\text{A}}^{pp}-{\text{A}}^{pn}{\left({\text{A}}^{nn}\right)}^{-1}{\text{A}}^{np}={\text{A}}_{pp}^{-1}\\ \Rightarrow \Phi ={\text{A}}_{pp}^{-1} \end{array}$$

Thus, forming and solving the mixed model equations using **Φ** will produce the same results as forming and solving the mixed model equations using ${\text{A}}_{pp}^{-1}$, but without the need to form **A**, invert or store **A**_*pp*_.

We carried out a test to compare the computational time between the full and the reduced animal model. Pedigree and phenotypes of 10,000 animals were simulated using R package pedSimulate (Nilforooshan, 2021). The pedigree was initiated with 100 founder animals (generation 0) of both sexes (50 each) and continued to eight generations. Except 2,000 randomly selected phenotypes from generations 6 to 8, other phenotypes were set to missing. Phenotypes were randomly assigned to five herds and the herd effect was added to phenotypes. The R statistical software (R Core Team, 2019) was used to analyse the data.

### 2.2. Multiple Trait Animal Model

The mixed model equations for a multiple trait animal model in its simplest form with no additional random effects other than for breeding values can be represented as:

(8) $$\begin{array}{l} \left[\begin{array}{cc} {{\text{X}}^{\prime }\text{R}}^{-1}\text{X} & {{\text{X}}^{\prime }\text{R}}^{-1}\text{Z}\\ {{\text{Z}}^{\prime }\text{R}}^{-1}\text{X} & {{\text{Z}}^{\prime }\text{R}}^{-1}\text{Z}+{\text{A}}^{-1}⊗{\text{G}}^{-1} \end{array}\right]\left[\begin{array}{c} \hat{\text{b}}\\ \hat{\text{u}} \end{array}\right]=\left[\begin{array}{c} {\text{X}}^{\prime }{\text{R}}^{-1}\text{y}\\ {{\text{Z}}^{\prime }\text{R}}^{-1}\text{y} \end{array}\right], \end{array}$$

where **G** is the *t* × *t* matrix of genetic covariances between the traits, **R** is the matrix of residual covariances between all the residuals on all the phenotyped animals, and ⊗ is the Kronecker product (Horn and Johnson, 1991). Permuting rows and columns of **A**^−1^ so that animals that are non-phenotyped (for all the traits) appear before animals that are phenotyped for at least one of the traits, $\text{Z}=\left[\begin{array}{cc} 0 & {\text{Z}}_{p} \end{array}\right]$, **Z**_*p*_ corresponds to animals phenotyped for at least one of the traits, and:

(9) $$\begin{array}{l} \left[\begin{array}{ccc} {{\text{X}}^{\prime }\text{R}}^{-1}\text{X} & 0 & {{\text{X}}^{\prime }\text{R}}^{-1}{\text{Z}}_{p}\\ 0 & {\text{A}}^{nn}⊗{\text{G}}^{-1} & {\text{A}}^{np}⊗{\text{G}}^{-1}\\ {\text{Z}}_{p}^{\prime }{\text{R}}^{-1}\text{X} & {\text{A}}^{pn}⊗{\text{G}}^{-1} & {\text{Z}}_{p}^{\prime }{\text{R}}^{-1}{\text{Z}}_{p}+{\text{A}}^{pp}⊗{\text{G}}^{-1} \end{array}\right]\left[\begin{array}{c} \hat{\text{b}}\\ {\hat{\text{u}}}_{n}\\ {\hat{\text{u}}}_{p} \end{array}\right]\\ \begin{array}{l} \;=\left[\begin{array}{c} {{\text{X}}^{\prime }\text{R}}^{-1}\text{y}\\ 0\\ {\text{Z}}_{p}^{\prime }{\text{R}}^{-1}\text{y} \end{array}\right]. \end{array} \end{array}$$

In the case of a 2-trait model, $\text{X}=\left[\begin{array}{cc} {\text{X}}_{1} & 0\\ 0 & {\text{X}}_{2} \end{array}\right]\text{ and }{\text{Z}}_{p}=\left[\begin{array}{cc} {\text{Z}}_{p_{1}} & 0\\ 0 & {\text{Z}}_{p_{2}} \end{array}\right].$ **Z**_*p*_1__ and **Z**_*p*_2__ contain columns for the same set of animals (those phenotyped for either or both of the two traits).

Following the same procedure as for the single-trait animal model, the model is reduced to fitting phenotyped animals for at least one of the traits in the model:

(10) $$\begin{array}{l} \left[\begin{array}{cc} {{\text{X}}^{\prime }\text{R}}^{-1}\text{X} & {{\text{X}}^{\prime }\text{R}}^{-1}{\text{Z}}_{p}\\ {\text{Z}}_{p}^{\prime }{\text{R}}^{-1}\text{X} & {\text{Z}}_{p}^{\prime }{\text{R}}^{-1}{\text{Z}}_{p}+\Phi ⊗{\text{G}}^{-1} \end{array}\right]\left[\begin{array}{c} \hat{\text{b}}\\ {\hat{\text{u}}}_{p} \end{array}\right]=\left[\begin{array}{c} {{\text{X}}^{\prime }\text{R}}^{-1}\text{y}\\ {\text{Z}}_{p}^{\prime }{\text{R}}^{-1}\text{y} \end{array}\right]. \end{array}$$

Considering Equation (9), solutions for non-phenotyped animals $\left({\hat{\text{u}}}_{n}\right)$ can be back-solved as:

(11) $$\begin{array}{l} \left({\text{A}}^{nn}⊗{\text{G}}^{-1}\right){\hat{\text{u}}}_{n}=-\left({\text{A}}^{np}⊗{\text{G}}^{-1}\right){\hat{\text{u}}}_{p}. \end{array}$$

### 2.3. Repeatability Animal Model

Repeatability animal models are employed for the analysis of repeated phenotypes of the same trait on an animal. In the simplest repeatability animal models, a genetic correlation of 1 and equal residual correlations are considered between repeated records from an animal, and the same variances are applied to all the phenotypes (Mrode, 2005). Repeatability animal models are typically fitted including an additional random term for permanent environmental effects (**y** = **Xb** + **Zu** + **Wp** + **e**). In matrix notation:

(12) $$\begin{array}{l} \left[\begin{array}{ccc} {\text{X}}^{\prime }\text{X} & {\text{X}}^{\prime }\text{Z} & {\text{X}}^{\prime }\text{W}\\ {\text{Z}}^{\prime }\text{X} & {\text{Z}}^{\prime }\text{Z}+\lambda {\text{A}}^{-1} & {\text{Z}}^{\prime }\text{W}\\ {\text{W}}^{\prime }\text{X} & {\text{W}}^{\prime }\text{Z} & {\text{W}}^{\prime }\text{W}+\alpha \text{I} \end{array}\right]\left[\begin{array}{c} \hat{\text{b}}\\ \hat{\text{u}}\\ \hat{\text{p}} \end{array}\right]=\left[\begin{array}{c} {\text{X}}^{\prime }\text{y}\\ {\text{Z}}^{\prime }\text{y}\\ {\text{W}}^{\prime }\text{y} \end{array}\right], \end{array}$$

where $\hat{\text{p}}$ is the vector of solutions for random permanent environmental effects, **W** is the incidence matrix relating phenotypes to permanent environmental effects), $\alpha =\sigma_{e}^{2}/\sigma_{p}^{2}$, and $\sigma_{p}^{2}$ is the permanent environment variance. Considering **W** being already limited to phenotyped animals, **W** = **Z**_*p*_, and:

(13) $$\begin{array}{l} \left[\begin{array}{cccc} {\text{X}}^{\prime }\text{X} & 0 & {{\text{X}}^{\prime }\text{Z}}_{p} & {\text{X}}^{\prime }\text{W}\\ 0 & \lambda {\text{A}}^{nn} & \lambda {\text{A}}^{np} & 0\\ {\text{Z}}_{p}^{\prime }\text{X} & \lambda {\text{A}}^{pn} & {\text{Z}}_{p}^{\prime }{\text{Z}}_{p}+\lambda {\text{A}}^{pp} & {\text{Z}}_{p}^{\prime }\text{W}\\ {\text{W}}^{\prime }\text{X} & 0 & {{\text{W}}^{\prime }\text{Z}}_{p} & {\text{W}}^{\prime }\text{W}+\alpha \text{I} \end{array}\right]\left[\begin{array}{c} \hat{\text{b}}\\ {\hat{\text{u}}}_{n}\\ {\hat{\text{u}}}_{p}\\ \hat{\text{p}} \end{array}\right]=\left[\begin{array}{c} {\text{X}}^{\prime }\text{y}\\ 0\\ {\text{Z}}_{p}^{\prime }\text{y}\\ {\text{W}}^{\prime }\text{y} \end{array}\right]. \end{array}$$

Equation (13) can be reduced to phenotyped animals (Equation 14) by absorbing the equations corresponding to breeding values for non-phenotyped animals into the equations corresponding to breeding values for phenotyped animals:

(14) $$\begin{array}{l} \left[\begin{array}{ccc} {\text{X}}^{\prime }\text{X} & {{\text{X}}^{\prime }\text{Z}}_{p} & {\text{X}}^{\prime }\text{W}\\ {\text{Z}}_{p}^{\prime }\text{X} & {\text{Z}}_{p}^{\prime }{\text{Z}}_{p}+\lambda \Phi & {\text{Z}}_{p}^{\prime }\text{W}\\ {\text{W}}^{\prime }\text{X} & {{\text{W}}^{\prime }\text{Z}}_{p} & {\text{W}}^{\prime }\text{W}+\alpha \text{I} \end{array}\right]\left[\begin{array}{c} \hat{\text{b}}\\ {\hat{\text{u}}}_{p}\\ \hat{\text{p}} \end{array}\right]=\left[\begin{array}{c} {\text{X}}^{\prime }\text{y}\\ {\text{Z}}_{p}^{\prime }\text{y}\\ {\text{W}}^{\prime }\text{y} \end{array}\right]. \end{array}$$

Back-solving solutions for non-phenotyped animals is done according to Equation (6). If permanent environmental effect solutions are not of interest, Equation (14) can be further reduced by absorbing the equations for random permanent environmental effects. Any random effect not correlated to other random effects remaining in the model can be confounded with the residual term (Quaas and Pollak, 1980).

### 2.4. Maternal Trait Animal Models

For some traits, dams not only influence the phenotypic expression of their progeny via directly passed genes, but also through maternal ability that influences the environment for their progeny, such as the milk volume they provide to the offspring (Mrode, 2005). Maternal ability is partly genetic and partly environmental. Accordingly, there can be animal models with maternal genetic effects, maternal permanent environment effects, or both. Animal models with maternal permanent environment effects have similar structure to repeatability animal models with the differences that there might be no repeated records per animal, **W** is the incidence matrix relating phenotypes to the dams of phenotyped animals, and a different variance than $\sigma_{p}^{2}$ is involved ($\sigma_{mpe}^{2}$, maternal permanent environment variance). Maternal permanent environmental effect is already limited to phenotyped animals as the incidence matrix used for this effect has rows corresponding to phenotypes and columns corresponding to dams of phenotyped animals.

Animal models with maternal genetic effects (**y** = **Xb** + **Zu** + **Sm** + **e**) uncorrelated to direct additive genetic effects have the variance structure:

$$\begin{array}{l} var\left[\begin{array}{c} \text{u}\\ \text{m}\\ \text{e} \end{array}\right]=\left[\begin{array}{ccc} \text{A}\sigma_{u}^{2} & 0 & 0\\ 0 & \text{A}\sigma_{m}^{2} & 0\\ 0 & 0 & \text{I}\sigma_{e}^{2} \end{array}\right], \end{array}$$

where $\sigma_{m}^{2}$ is the maternal genetic variance. In matrix notation:

(15) $$\begin{array}{l} \left[\begin{array}{ccc} {\text{X}}^{\prime }\text{X} & {\text{X}}^{\prime }\text{Z} & {\text{X}}^{\prime }\text{S}\\ {\text{Z}}^{\prime }\text{X} & {\text{Z}}^{\prime }\text{Z}+\lambda_{1}{\text{A}}^{-1} & {\text{Z}}^{\prime }\text{S}\\ {\text{S}}^{\prime }\text{X} & {\text{S}}^{\prime }\text{Z} & {\text{S}}^{\prime }\text{S}+\lambda_{2}{\text{A}}^{-1} \end{array}\right]\left[\begin{array}{c} \hat{\text{b}}\\ \hat{\text{u}}\\ \hat{\text{m}} \end{array}\right]=\left[\begin{array}{c} {\text{X}}^{\prime }\text{y}\\ {\text{Z}}^{\prime }\text{y}\\ {\text{S}}^{\prime }\text{y} \end{array}\right]. \end{array}$$

Given $\text{Z}=\left[\begin{array}{cc} 0 & {\text{Z}}_{p} \end{array}\right],\text{S}=\left[\begin{array}{cc} {\text{S}}_{n} & {\text{S}}_{p} \end{array}\right]\text{ and }S^{\prime }S=\left[\begin{array}{cc} {S^{\prime }}_{n}{\text{S}}_{n} & 0\\ 0 & {S^{\prime }}_{p}{\text{S}}_{p} \end{array}\right]$, Equation (15) can be re-written as:

(16) $$\begin{array}{l} \left[\begin{array}{ccccc} {\text{X}}^{\prime }\text{X} & 0 & {{\text{X}}^{\prime }\text{Z}}_{p} & {{\text{X}}^{\prime }\text{S}}_{n} & {{\text{X}}^{\prime }\text{S}}_{p}\\ 0 & \lambda_{1}{\text{A}}^{nn} & \lambda_{1}{\text{A}}^{np} & 0 & 0\\ {\text{Z}}_{p}^{\prime }\text{X} & \lambda_{1}{\text{A}}^{pn} & {\text{Z}}_{p}^{\prime }{\text{Z}}_{p}+\lambda_{1}{\text{A}}^{pp} & {\text{Z}}_{p}^{\prime }{\text{S}}_{n} & {\text{Z}}_{p}^{\prime }{\text{S}}_{p}\\ {\text{S}}_{n}^{\prime }\text{X} & 0 & {\text{S}}_{n}^{\prime }{\text{Z}}_{p} & {\text{S}}_{n}^{\prime }{\text{S}}_{n}+\lambda_{2}{\text{A}}^{nn} & \lambda_{2}{\text{A}}^{np}\\ {\text{S}}_{p}^{\prime }\text{X} & 0 & {\text{S}}_{p}^{\prime }{\text{Z}}_{p} & \lambda_{2}{\text{A}}^{pn} & {\text{S}}_{p}^{\prime }{\text{S}}_{p}+\lambda_{2}{\text{A}}^{pp} \end{array}\right]\\ \begin{array}{l} \;[\begin{array}{c} \hat{\text{b}}\\ {\hat{\text{u}}}_{n}\\ {\hat{\text{u}}}_{p}\\ {\hat{\text{m}}}_{n}\\ {\hat{\text{m}}}_{p} \end{array}]=\left[\begin{array}{c} {\text{X}}^{\prime }\text{y}\\ 0\\ {\text{Z}}_{p}^{\prime }\text{y}\\ 0\\ {\text{S}}_{p}^{\prime }\text{y} \end{array}\right], \end{array} \end{array}$$

where λ_1_ and λ_2_ are equal to $\sigma_{e}^{2}/\sigma_{u}^{2}$ and $\sigma_{e}^{2}/\sigma_{m}^{2}$, respectively. Considering equations corresponding to ${\hat{\text{u}}}_{n}$ in Equation (16), Equation (6) is derived. Equation (16) can then be reduced to:

(17) $$\begin{array}{l} \left[\begin{array}{ccc} {\text{X}}^{\prime }\text{X} & {{\text{X}}^{\prime }\text{Z}}_{p} & {\text{X}}^{\prime }\text{S}\\ {\text{Z}}_{p}^{\prime }\text{X} & {\text{Z}}_{p}^{\prime }{\text{Z}}_{p}+\lambda_{1}\Phi & {\text{Z}}_{p}^{\prime }\text{S}\\ {\text{S}}^{\prime }\text{X} & {{\text{S}}^{\prime }\text{Z}}_{p} & {\text{S}}^{\prime }\text{S}+\lambda_{2}{\text{A}}^{-1} \end{array}\right]\left[\begin{array}{c} \hat{\text{b}}\\ {\hat{\text{u}}}_{p}\\ \hat{\text{m}} \end{array}\right]=\left[\begin{array}{c} {\text{X}}^{\prime }\text{y}\\ {\text{Z}}_{p}^{\prime }\text{y}\\ {\text{S}}^{\prime }\text{y} \end{array}\right]. \end{array}$$

Equations corresponding to $\hat{\text{m}}$ cannot be reduced to equations corresponding to ${\hat{\text{m}}}_{p}$. The reason is the non-zero **S**_*n*_ matrix.

Animal models with maternal genetic effects correlated to direct additive genetic effects have the variance structure:

$$\begin{array}{l} var\left[\begin{array}{c} \text{u}\\ \text{m}\\ \text{e} \end{array}\right]=\left[\begin{array}{ccc} \text{A}\sigma_{u}^{2} & \text{A}\sigma_{um} & 0\\ \text{A}\sigma_{mu} & \text{A}\sigma_{m}^{2} & 0\\ 0 & 0 & \text{I}\sigma_{e}^{2} \end{array}\right]. \end{array}$$

In matrix notation:

(18) $$\begin{array}{l} \left[\begin{array}{ccc} {\text{X}}^{\prime }\text{X} & {\text{X}}^{\prime }\text{Z} & {\text{X}}^{\prime }\text{S}\\ {\text{Z}}^{\prime }\text{X} & {\text{Z}}^{\prime }\text{Z}+\lambda_{11}{\text{A}}^{-1} & {\text{Z}}^{\prime }\text{S}+\lambda_{12}{\text{A}}^{-1}\\ {\text{S}}^{\prime }\text{X} & {\text{S}}^{\prime }\text{Z}+\lambda_{21}{\text{A}}^{-1} & {\text{S}}^{\prime }\text{S}+\lambda_{22}{\text{A}}^{-1} \end{array}\right]\left[\begin{array}{c} \hat{\text{b}}\\ \hat{\text{u}}\\ \hat{\text{m}} \end{array}\right]=\left[\begin{array}{c} {\text{X}}^{\prime }\text{y}\\ {\text{Z}}^{\prime }\text{y}\\ {\text{S}}^{\prime }\text{y} \end{array}\right], \end{array}$$

where $\left[\begin{array}{cc} \lambda_{11} & \lambda_{12}\\ \lambda_{21} & \lambda_{22} \end{array}\right]=\sigma_{e}^{2}{\text{G}}^{-1}$, and ${\text{G}}^{-1}=\left[\begin{array}{cc} g^{11} & g^{12}\\ g^{21} & g^{22} \end{array}\right]={\left[\begin{array}{cc} \sigma_{u}^{2} & \sigma_{um}\\ \sigma_{mu} & \sigma_{m}^{2} \end{array}\right]}^{-1}$.

For an efficient model reduction, it is important that all **A**^−1^ occurrences in Equation (18) are converted to the same matrix. In order to do that, the definition of *n* and *p* are changed to *p* being phenotyped animals or non-phenotyped dams, and *n* being other animals. That enables both **Z**_*n*_ and **S**_*n*_ being zero matrices. Then, permuting rows and columns of **A**^−1^ so that animals *n* appear before animals *p*, Equation (18) can be written as:

(19) $$\begin{array}{l} \left[\begin{array}{ccccc} {\text{X}}^{\prime }\text{X} & 0 & {{\text{X}}^{\prime }\text{Z}}_{p} & 0 & {{\text{X}}^{\prime }\text{S}}_{p}\\ 0 & \lambda_{11}{\text{A}}^{nn} & \lambda_{11}{\text{A}}^{np} & \lambda_{12}{\text{A}}^{nn} & \lambda_{12}{\text{A}}^{np}\\ {\text{Z}}_{p}^{\prime }\text{X} & \lambda_{11}{\text{A}}^{pn} & {\text{Z}}_{p}^{\prime }{\text{Z}}_{p}+\lambda_{11}{\text{A}}^{pp} & \lambda_{12}{\text{A}}^{pn} & {\text{Z}}_{p}^{\prime }{\text{S}}_{p}+\lambda_{12}{\text{A}}^{pp}\\ 0 & \lambda_{21}{\text{A}}^{nn} & \lambda_{21}{\text{A}}^{np} & \lambda_{22}{\text{A}}^{nn} & \lambda_{22}{\text{A}}^{np}\\ {\text{S}}_{p}^{\prime }\text{X} & \lambda_{21}{\text{A}}^{pn} & {\text{S}}_{p}^{\prime }{\text{Z}}_{p}+\lambda_{21}{\text{A}}^{pp} & \lambda_{22}{\text{A}}^{pn} & {\text{S}}_{p}^{\prime }{\text{S}}_{p}+\lambda_{22}{\text{A}}^{pp} \end{array}\right]\\ \begin{array}{l} \;[\begin{array}{c} \hat{\text{b}}\\ {\hat{\text{u}}}_{n}\\ {\hat{\text{u}}}_{p}\\ {\hat{\text{m}}}_{n}\\ {\hat{\text{m}}}_{p} \end{array}]=\left[\begin{array}{c} {\text{X}}^{\prime }\text{y}\\ 0\\ {\text{Z}}_{p}^{\prime }\text{y}\\ 0\\ {\text{S}}_{p}^{\prime }\text{y} \end{array}\right]. \end{array} \end{array}$$

Reducing the above equation to phenotyped animals, equations corresponding to ${\hat{\text{u}}}_{n}$ are absorbed in equations corresponding to ${\hat{\text{u}}}_{p}$, and equations corresponding to ${\hat{\text{m}}}_{n}$ are absorbed in equations corresponding to ${\hat{\text{m}}}_{p}$, resulting to:

(20) $$\begin{array}{l} \left[\begin{array}{ccc} {\text{X}}^{\prime }\text{X} & {{\text{X}}^{\prime }\text{Z}}_{p} & {{\text{X}}^{\prime }\text{S}}_{p}\\ {\text{Z}}_{p}^{\prime }\text{X} & {\text{Z}}_{p}^{\prime }{\text{Z}}_{p}+\lambda_{11}\Phi & {\text{Z}}_{p}^{\prime }{\text{S}}_{p}+\lambda_{12}\Phi \\ {\text{S}}_{p}^{\prime }\text{X} & {\text{S}}_{p}^{\prime }{\text{Z}}_{p}+\lambda_{21}\Phi & {\text{S}}_{p}^{\prime }{\text{S}}_{p}+\lambda_{22}\Phi \end{array}\right]\left[\begin{array}{c} \hat{\text{b}}\\ {\hat{\text{u}}}_{p}\\ {\hat{\text{m}}}_{p} \end{array}\right]=\left[\begin{array}{c} {\text{X}}^{\prime }\text{y}\\ {\text{Z}}_{p}^{\prime }\text{y}\\ {\text{S}}_{p}^{\prime }\text{y} \end{array}\right]. \end{array}$$

${\hat{\text{u}}}_{n}$ and ${\hat{\text{m}}}_{n}$ are back-solved given ${\hat{\text{u}}}_{p}$, ${\hat{\text{m}}}_{p}$, **A**^*nn*^, **A**^*np*^, and **G**^−1^. For the proof, consider rows 2 and 4 in Equation (19):

$$\begin{array}{l} \;\{\begin{array}{c} \lambda_{11}{\text{A}}^{nn}{\hat{\text{u}}}_{n}+\lambda_{11}{\text{A}}^{np}{\hat{\text{u}}}_{p}+\lambda_{12}{\text{A}}^{nn}{\hat{\text{m}}}_{n}+\lambda_{12}{\text{A}}^{np}{\hat{\text{m}}}_{p}=0\\ \lambda_{21}{\text{A}}^{nn}{\hat{\text{u}}}_{n}+\lambda_{21}{\text{A}}^{np}{\hat{\text{u}}}_{p}+\lambda_{22}{\text{A}}^{nn}{\hat{\text{m}}}_{n}+\lambda_{22}{\text{A}}^{np}{\hat{\text{m}}}_{p}=0 \end{array}\\ \Rightarrow \{\begin{array}{c} {\text{A}}^{nn}(\lambda_{11}{\hat{\text{u}}}_{n}+\lambda_{12}{\hat{\text{m}}}_{n})=-{\text{A}}^{np}(\lambda_{11}{\hat{\text{u}}}_{p}+\lambda_{12}{\hat{\text{m}}}_{p})\\ {\text{A}}^{nn}(\lambda_{21}{\hat{\text{u}}}_{n}+\lambda_{22}{\hat{\text{m}}}_{n})=-{\text{A}}^{np}(\lambda_{21}{\hat{\text{u}}}_{p}+\lambda_{22}{\hat{\text{m}}}_{p}) \end{array}\\ \Rightarrow \{\begin{array}{c} {\text{A}}^{nn}(g^{11}{\hat{\text{u}}}_{n}+g^{12}{\hat{\text{m}}}_{n})=-{\text{A}}^{np}(g^{11}{\hat{\text{u}}}_{p}+g^{12}{\hat{\text{m}}}_{p})\\ {\text{A}}^{nn}(g^{21}{\hat{\text{u}}}_{n}+g^{22}{\hat{\text{m}}}_{n})=-{\text{A}}^{np}(g^{21}{\hat{\text{u}}}_{p}+g^{22}{\hat{\text{m}}}_{p}) \end{array} \end{array}$$

(21) $$\Rightarrow ({\text{A}}^{nn}⊗{\text{G}}^{-1})\left[\begin{array}{c} {\hat{\text{u}}}_{n}\\ {\hat{\text{m}}}_{n} \end{array}\right]=-({\text{A}}^{np}⊗{\text{G}}^{-1})\left[\begin{array}{c} {\hat{\text{u}}}_{p}\\ {\hat{\text{m}}}_{p} \end{array}\right]$$

### 2.5. Animal Models With Genetic Groups

The idea of sire models with genetic groups (Quaas and Pollak, 1981) was extended to animal models by Westell and Van Vleck (1987), where grouping stems from the fact that unknown parents belonging to different genetic backgrounds and periods of time have different genetic means. An animal model with genetic groups and no additional random effect other than breeding values is written as **y** = **Xb** + **Zu** + **ZQg** + **e**. In matrix notation:

(22) $$\begin{array}{l} \left[\begin{array}{ccc} {\text{X}}^{\prime }\text{X} & {\text{X}}^{\prime }\text{Z} & {\text{X}}^{\prime }Z\text{Q}\\ {\text{Z}}^{\prime }\text{X} & {\text{Z}}^{\prime }\text{Z}+\lambda {\text{A}}^{-1} & {\text{Z}}^{\prime }Z\text{Q}\\ {\text{Q}}^{\prime }Z^{\prime }\text{X} & {\text{Q}}^{\prime }Z^{\prime }\text{Z} & {\text{Q}}^{\prime }Z^{\prime }Z\text{Q} \end{array}\right]\left[\begin{array}{c} \hat{\text{b}}\\ \hat{\text{u}}\\ \hat{\text{g}} \end{array}\right]=\left[\begin{array}{c} {\text{X}}^{\prime }\text{y}\\ {\text{Z}}^{\prime }\text{y}\\ {\text{Q}}^{\prime }Z^{\prime }\text{y} \end{array}\right], \end{array}$$

where $\hat{\text{g}}$ is the vector of solutions for genetic groups and **Q** is the matrix relating genetic groups to animals. Permuting rows and columns of **A**^−1^ so that non-phenotyped animals appear before phenotyped animals and phenotyped animals appear in the same order in **y** and **A**^*pp*^, **Z** = $\text{Z}=\left[\begin{array}{cc} 0 & {\text{Z}}_{p} \end{array}\right]$, ${\text{Q}}^{\prime }=\left[\begin{array}{cc} {\text{Q}}_{n}^{\prime } & {\text{Q}}_{p}^{\prime } \end{array}\right]$, **ZQ** = **Z**_*p*_**Q**_*p*_, and **Z**_*p*_ = **I**. Thus, Equation (22) can be written as:

(23) $$\begin{array}{l} \left[\begin{array}{cccc} {\text{X}}^{\prime }\text{X} & 0 & {\text{X}}^{\prime } & {{\text{X}}^{\prime }\text{Q}}_{p}\\ 0 & \lambda {\text{A}}^{nn} & \lambda {\text{A}}^{np} & 0\\ \text{X} & \lambda {\text{A}}^{pn} & \text{I}+\lambda {\text{A}}^{pp} & {\text{Q}}_{p}\\ {\text{Q}}_{p}^{\prime }\text{X} & 0 & {\text{Q}}_{p}^{\prime } & {\text{Q}}_{p}^{\prime }{\text{Q}}_{p} \end{array}\right]\left[\begin{array}{c} \hat{\text{b}}\\ {\hat{\text{u}}}_{n}\\ {\hat{\text{u}}}_{p}\\ \hat{\text{g}} \end{array}\right]=\left[\begin{array}{c} {\text{X}}^{\prime }\text{y}\\ 0\\ \text{y}\\ {\text{Q}}_{p}^{\prime }\text{y} \end{array}\right]. \end{array}$$

Absorbing the equations corresponding to ${\hat{\text{u}}}_{n}$ in the equations corresponding to ${\hat{\text{u}}}_{p}$, the above model is reduced to:

(24) $$\begin{array}{l} \left[\begin{array}{ccc} {\text{X}}^{\prime }\text{X} & {\text{X}}^{\prime } & {{\text{X}}^{\prime }\text{Q}}_{p}\\ \text{X} & \text{I}+\lambda \Phi & {\text{Q}}_{p}\\ {\text{Q}}_{p}^{\prime }\text{X} & {\text{Q}}_{p}^{\prime } & {\text{Q}}_{p}^{\prime }{\text{Q}}_{p} \end{array}\right]\left[\begin{array}{c} \hat{\text{b}}\\ {\hat{\text{u}}}_{p}\\ \hat{\text{g}} \end{array}\right]=\left[\begin{array}{c} {\text{X}}^{\prime }\text{y}\\ \text{y}\\ {\text{Q}}_{p}^{\prime }\text{y} \end{array}\right]. \end{array}$$

Back-solving ${\hat{\text{u}}}_{n}$ is done using Equation (6) and the breeding value of animals are calculated as $\hat{\text{u}}+\text{Q}\hat{\text{g}}$. Forming **Q** to calculate breeding values outside of the animal model is an additional computational step. To avoid that step, Quaas and Pollak (1981) transformed the mixed model equations so that the solution for the breeding value includes the genetic group effect:

(25) $$\begin{array}{l} \left[\begin{array}{ccc} {\text{X}}^{\prime }\text{X} & {\text{X}}^{\prime }\text{Z} & 0\\ {\text{Z}}^{\prime }\text{X} & {\text{Z}}^{\prime }\text{Z}+\lambda {\text{A}}^{-1} & -\lambda {\text{A}}^{-1}\text{Q}\\ 0 & -\lambda {\text{Q}}^{\prime }{\text{A}}^{-1} & \lambda {\text{Q}}^{\prime }{\text{A}}^{-1}\text{Q} \end{array}\right]\left[\begin{array}{c} \hat{\text{b}}\\ \hat{\text{u}}+\text{Q}\hat{\text{g}}\\ \hat{\text{g}} \end{array}\right]=\left[\begin{array}{c} {\text{X}}^{\prime }\text{y}\\ {\text{Z}}^{\prime }\text{y}\\ 0 \end{array}\right]. \end{array}$$

Let's consider:

$$\begin{array}{l} \Lambda^{-1}=\left[\begin{array}{cc} {\text{A}}^{-1} & -{\text{A}}^{-1}\text{Q}\\ -{\text{Q}}^{\prime }{\text{A}}^{-1} & {\text{Q}}^{\prime }{\text{A}}^{-1}\text{Q} \end{array}\right]=\left[\begin{array}{cc} {\text{A}}^{-1} & {\text{A}}^{npg}\\ {\text{A}}^{gpn} & {\text{A}}^{gg} \end{array}\right]\\ \;=\left[\begin{array}{ccc} {\text{A}}^{nn} & {\text{A}}^{np} & {\text{A}}^{ng}\\ {\text{A}}^{pn} & {\text{A}}^{pp} & {\text{A}}^{pg}\\ {\text{A}}^{gn} & {\text{A}}^{gp} & {\text{A}}^{gg} \end{array}\right], \end{array}$$

where *g* corresponds to genetic groups in a larger numerator relationship matrix (i.e., **Λ** is larger than **A**). Then, Equation (25) can be written as:

$$\begin{array}{l} \left[\begin{array}{cccc} {\text{X}}^{\prime }\text{X} & 0 & {\text{X}}^{\prime }{\text{Z}}_{p} & 0\\ 0 & \lambda {\text{A}}^{nn} & \lambda {\text{A}}^{np} & \lambda {\text{A}}^{ng}\\ {\text{Z}}_{p}^{\prime }\text{X} & \lambda {\text{A}}^{pn} & {\text{Z}}_{p}^{\prime }{\text{Z}}_{p}+\lambda {\text{A}}^{pp} & \lambda {\text{A}}^{pg}\\ 0 & \lambda {\text{A}}^{gn} & \lambda {\text{A}}^{gp} & \lambda {\text{A}}^{gg} \end{array}\right]\left[\begin{array}{c} \hat{\text{b}}\\ {\hat{\text{u}}}_{n}+{\text{Q}}_{n}\hat{\text{g}}\\ {\hat{\text{u}}}_{p}+{\text{Q}}_{p}\hat{\text{g}}\\ \hat{\text{g}} \end{array}\right]=\left[\begin{array}{c} {\text{X}}^{\prime }\text{y}\\ 0\\ {\text{Z}}_{p}^{\prime }\text{y}\\ 0 \end{array}\right]. \end{array}$$

Considering **Z**_*pg*_ = ${\text{Z}}_{pg}=\left[\begin{array}{cc} {\text{Z}}_{p} & 0 \end{array}\right]$, in which **0** is a matrix of zeros with columns corresponding to genetic groups:

(26) $$\begin{array}{l} \left[\begin{array}{ccc} X^{\prime }X & 0 & X^{\prime }{\text{Z}}_{pg}\\ 0 & \lambda {\text{A}}^{nn} & \lambda \left[\begin{array}{cc} {\text{A}}^{np} & {\text{A}}^{ng} \end{array}\right]\\ {Z^{\prime }}_{pg}\text{X} & \lambda \left[\begin{array}{c} {\text{A}}^{pn}\\ {\text{A}}^{gn} \end{array}\right] & {Z^{\prime }}_{pg}{\text{Z}}_{pg}+\lambda \left[\begin{array}{cc} {\text{A}}^{pp} & {\text{A}}^{pg}\\ {\text{A}}^{gp} & {\text{A}}^{gg} \end{array}\right] \end{array}\right]\left[\begin{array}{c} \hat{\text{b}}\\ {\hat{\text{u}}}_{n}+{\text{Q}}_{n}\hat{\text{g}}\\ {\hat{\text{u}}}_{p}+{\text{Q}}_{p}\hat{\text{g}}\\ \hat{\text{g}} \end{array}\right]\\ \;=\left[\begin{array}{c} X^{\prime }y\\ 0\\ {Z^{\prime }}_{pg}\text{y} \end{array}\right]. \end{array}$$

Applying the same technique used to reduce Equation (3) to Equation (4), Equation (26) is reduced to Equation (27):

(27) $$\begin{array}{l} \left[\begin{array}{cc} {\text{X}}^{\prime }\text{X} & {{\text{X}}^{\prime }\text{Z}}_{pg}\\ {\text{Z}}_{pg}^{\prime }\text{X} & {\text{Z}}_{pg}^{\prime }{\text{Z}}_{pg}+\lambda \Phi \end{array}\right]\left[\begin{array}{c} \hat{\text{b}}\\ {\hat{\text{u}}}_{p}+{\text{Q}}_{p}\hat{\text{g}}\\ \hat{\text{g}} \end{array}\right]=\left[\begin{array}{c} {\text{X}}^{\prime }\text{y}\\ {\text{Z}}_{pg}^{\prime }\text{y} \end{array}\right], \end{array}$$

where extending Equation (5) to include genetic groups:

(28) $$\begin{array}{l} \Phi =\left[\begin{array}{cc} \Phi_{pp} & \Phi_{pg}\\ \Phi_{gp} & \Phi_{gg} \end{array}\right]=\left[\begin{array}{cc} {\text{A}}^{pp} & {\text{A}}^{pg}\\ {\text{A}}^{gp} & {\text{A}}^{gg} \end{array}\right]-\left[\begin{array}{c} {\text{A}}^{pn}\\ {\text{A}}^{gn} \end{array}\right]{\left({\text{A}}^{nn}\right)}^{-1}{\left[\begin{array}{c} {\text{A}}^{pn}\\ {\text{A}}^{gn} \end{array}\right]}^{\prime } \end{array}$$

and extending Equation (6) to include genetic groups:

(29) $${\text{A}}^{nn}({\hat{\text{u}}}_{n}+{\text{Q}}_{n}\hat{\text{g}})=-\left[\begin{array}{cc} {\text{A}}^{np} & {\text{A}}^{np} \end{array}\right]\left(\begin{array}{c} {\hat{\text{u}}}_{p}+{\text{Q}}_{p}\hat{\text{g}}\\ \hat{\text{g}} \end{array}\right).$$

### 2.6. Animal Models With a Genomic Relationship Matrix (GBLUP)

In the previous sections, animal models with pedigree-based relationship matrices were discussed. With the availability of genomic data, various forms of genomic relationship matrix (e.g., VanRaden, 2008; Hickey et al., 2013) are used in animal models. Replacing **A** with the genomic relationship matrix **G** in Equation (1) results in:

(30) $$\begin{array}{l} \left[\begin{array}{cc} {\text{X}}^{\prime }\text{X} & {\text{X}}^{\prime }\text{Z}\\ {\text{Z}}^{\prime }\text{X} & {\text{Z}}^{\prime }\text{Z}+\lambda {\text{G}}^{-1} \end{array}\right]\left[\begin{array}{c} \hat{\text{b}}\\ \hat{\text{u}} \end{array}\right]=\left[\begin{array}{c} {\text{X}}^{\prime }\text{y}\\ {\text{Z}}^{\prime }\text{y} \end{array}\right]. \end{array}$$

However, it should be noted that unlike Equation (1), Equation (30) is limited to genotyped animals. Due to efficient methods for deriving **A**^−1^ without calculating and inverting **A** (Henderson, 1976; Quaas, 1976), Equation (4) is preferred over Equation (7). However, those methods are not applicable to **G**, and this form of the equations requires **G** to be calculated and directly inverted. As a result, the reduced method of choice is equivalent to Equation (7):

(31) $$\begin{array}{l} \left[\begin{array}{cc} {\text{X}}^{\prime }\text{X} & {\text{X}}^{\prime }\\ \text{X} & \text{I}+\lambda {\text{G}}_{pp}^{-1} \end{array}\right]\left[\begin{array}{c} \hat{\text{b}}\\ {\hat{\text{u}}}_{p} \end{array}\right]=\left[\begin{array}{c} {\text{X}}^{\prime }\text{y}\\ \text{y} \end{array}\right]. \end{array}$$

Therefore, only **G**_*pp*_ needs to be formed and inverted. If the calculation of **G**_*pp*_ requires allele frequencies, estimation of allele frequencies should not be limited to phenotyped animals. Then, ${\hat{\text{u}}}_{n}$ can be derived indirectly by, first back-solving marker effects $\left(\hat{\text{a}}\right)$ from ${\hat{\text{u}}}_{p}$ (Strandén and Garrick, 2009), and then summing up marker effect solutions over all markers for non-phenotyped animals (${\hat{\text{u}}}_{n}={\text{M}}_{n}\hat{\text{a}}$ (Meuwissen et al., 2001), where **M**_*n*_ contains rows of **M** for non-phenotyped animals, and **M** is the genotype matrix with coefficients −1, 0, and 1).

### 2.7. Animal Models With an Augmented Pedigree-Genomic Relationship Matrix (ssGBLUP)

Aguilar et al. (2010) and Christensen and Lund (2010) introduced an animal model for simultaneous genetic evaluation of genotyped and non-genotyped animal, using a pedigree-genomic augmented inverse matrix (**H**^−1^) to be used instead of **A**^−1^ in the mixed model equations, where

(32) $$\begin{array}{l} {\text{H}}^{-1}=\left[\begin{array}{cc} {\text{H}}^{11} & {\text{H}}^{12}\\ {\text{H}}^{21} & {\text{H}}^{22} \end{array}\right]=\left[\begin{array}{cc} {\text{A}}^{11} & {\text{A}}^{12}\\ {\text{A}}^{21} & {\text{A}}^{22}+{\text{G}}^{-1}-{\text{A}}_{22}^{-1} \end{array}\right], \end{array}$$

**H**^11^ corresponds to the block for non-genotyped animals, and **H**^22^ corresponds to the block for genotyped animals. Though, it is possible to partition **H**^−1^ to $\left[\begin{array}{cc} {\text{H}}^{nn} & {\text{H}}^{np}\\ {\text{H}}^{pn} & {\text{H}}^{pp} \end{array}\right]$ and reduce the model to phenotyped animals (similar to the procedures already explained), perhaps a more practical approach would be to split animals into three groups of “non-phenotyped and non-genotyped” animals (4), “phenotyped and non-genotyped” animals (3), and genotyped animals (2):

$$\begin{array}{l} {\text{H}}^{-1}=\left[\begin{array}{ccc} {\text{H}}^{44} & {\text{H}}^{43} & {\text{H}}^{42}\\ {\text{H}}^{34} & {\text{H}}^{33} & {\text{H}}^{32}\\ {\text{H}}^{24} & {\text{H}}^{23} & {\text{H}}^{22} \end{array}\right]=\left[\begin{array}{ccc} {\text{A}}^{44} & {\text{A}}^{43} & {\text{A}}^{42}\\ {\text{A}}^{34} & {\text{A}}^{33} & {\text{A}}^{32}\\ {\text{A}}^{24} & {\text{A}}^{23} & {\text{H}}^{22} \end{array}\right]. \end{array}$$

Grouping phenotyped or genotyped animals together (5):

$$\begin{array}{l} {\text{H}}^{-1}=\left[\begin{array}{cc} {\text{A}}^{44} & {\text{A}}^{45}\\ {\text{A}}^{54} & {\text{H}}^{55} \end{array}\right],{\text{H}}^{55}=\left[\begin{array}{cc} {\text{A}}^{33} & {\text{A}}^{32}\\ {\text{A}}^{23} & {\text{H}}^{22} \end{array}\right],{\text{A}}^{54}=\left[\begin{array}{c} {\text{A}}^{34}\\ {\text{A}}^{24} \end{array}\right]. \end{array}$$

Then, an animal model like the following:

(33) $$\begin{array}{l} \left[\begin{array}{cc} {\text{X}}^{\prime }\text{X} & {\text{X}}^{\prime }\text{Z}\\ {\text{Z}}^{\prime }\text{X} & {\text{Z}}^{\prime }\text{Z}+\lambda {\text{H}}^{-1} \end{array}\right]\left[\begin{array}{c} \hat{\text{b}}\\ \hat{\text{u}} \end{array}\right]=\left[\begin{array}{c} {\text{X}}^{\prime }\text{y}\\ {\text{Z}}^{\prime }\text{y} \end{array}\right] \end{array}$$

can be reduced to:

(34) $$\begin{array}{l} \left[\begin{array}{cc} {\text{X}}^{\prime }\text{X} & {{\text{X}}^{\prime }\text{Z}}_{5}\\ {\text{Z}}_{5}^{\prime }\text{X} & {\text{Z}}_{5}^{\prime }{\text{Z}}_{5}+\lambda \Theta \end{array}\right]\left[\begin{array}{c} \hat{\text{b}}\\ {\hat{\text{u}}}_{5} \end{array}\right]=\left[\begin{array}{c} {\text{X}}^{\prime }\text{y}\\ {\text{Z}}_{5}^{\prime }\text{y} \end{array}\right], \end{array}$$

where **Θ** = **H**^55^ − **A**^54^(**A**^44^) ^− 1^**A**^45^ and **Z**_5_ contains columns of **Z** corresponding to group 5 of animals. After obtaining ${\hat{\text{u}}}_{5}$, ${\hat{\text{u}}}_{4}$ can be back-solved by solving

(35) $$\begin{array}{l} {\text{A}}^{44}{\hat{\text{u}}}_{4}=-{\text{A}}^{45}{\hat{\text{u}}}_{5}. \end{array}$$

## 3. Results

Results from numerical examples are provided. The numerical examples were chosen from small datasets used in Mrode (2005) for different animal models. R scripts for producing the data and the analyses are available in the data repository. Files for any given filename in this study are also available in the data repository. Back-solving were done by direct solving of Equations (6), (11), (21), and (29). Where solving these equations for large pedigree is computationally challenging, preconditioned conjugate gradient method (Strandén and Lidauer, 1999) can be adopted.

### 3.1. Basic Animal Model

Data from Example 3.1 of Mrode (2005) was used (Supplementary Table 1). The data can be produced using e31data.R in the data repository. Equation (1) is coded in function “BLUP” in e31.R. The following code produces the data and solutions.


# Create data
source(“e31data.R”)
# Call the solver function
source(“e31.R”)
# Solve the full model
sol1 = BLUP(X, y, Z, lambda, Ai)


Applying Equation (4) instead of Equation (1) requires data transformation. Matrices **Φ**, **A**^*nn*^ and **A**^*np*^ are obtained using function “getPhi” coded in getPhi.R. Solutions of the reduced model are obtained as:


# Call the function to create Phi matrix
source(“getPhi.R”)
# Define phenotyped animals
phe=4:8
# Create Phi and back-solving matrices
mats = getPhi(Ai,phe)
# Solve the reduced model
sol2 = BLUP(X, y, Z=Z[,phe], lambda,
 Ai=mats$Phi)


Following Equation (6), ${\hat{\text{u}}}_{n}$ are back-solved using the following code:


u_n = solve(mats$Ann, mats$Anp %*%
 -sol2[-2:-1])


The following command confirms that solutions from the full (Equation 1) and the reduced models (Equation 4) are identical.


source(“check.R”) # Call the function
check(sol1, c(sol2[1:2], u_n, sol2[3:7]))


We run a test to compare the computational time for the full and the reduced animal model for a pedigree of 10,000 animals, of which 2,000 animals had been phenotyped. The runtime were 10 and 8.8 s for the full and the reduced animal model, respectively. Additionally, it took 2 s for the reduced animal model to calculate **Φ**. Matrix **Φ** was considerably denser than the **A**^−1^ matrix.

### 3.2. Multiple Trait Animal Model

Data from Example 5.3 of Mrode (2005) was used (Supplementary Table 2). The data can be produced using e53data.R in the data repository. Equation (8) is coded in function “BLUP” in e53.R. The following code produces the data and solutions.


# Create data
source(“e53data.R”)
# Call the solver function
source(“e53.R”)
# Solve the full model
sol3 = BLUP(X, y, Z, Ri, Gi, Ai)


Applying Equation (10) instead of Equation (8) requires data transformation. Matrices **Φ**, **A**^*nn*^ and **A**^*np*^ are obtained using function “getPhi” coded in getPhi.R. Solutions of the reduced model are obtained as:


# Call the function to create Phi matrix
source(“getPhi.R”)
# Define phenotyped animals
phe=4:9
# Create Phi and back-solving matrices
mats = getPhi(Ai,phe)
# Solve the reduced model
sol4 = BLUP(X, y, Z=Z[,c(phe,phe+n)],
 Ri, Gi, Ai=mats$Phi)


Following Equation (11), ${\hat{\text{u}}}_{n}$ are back-solved using the following code:


u_n = solve(kronecker(Gi, mats$Ann),
     kronecker(Gi, mats$Anp) %*%
      -sol4[-4:-1])


The following command confirms that solutions from the full (Equation 8) and the reduced models (Equation 10) are identical.


source(“check.R”) # Call the function
check(sol3, c(sol4[1:4], u_n[1:3],
sol4[5:10], u_n[4:6], sol4[11:16]))


### 3.3. Repeatability Animal Model

Data from Example 4.1 of Mrode (2005) was used (Supplementary Table 3). The data can be produced using e41data.R in the data repository. Equation (12) is coded in function “BLUP” in e41.R. The following code produces the data and solutions.


# Create data
source(“e41data.R”)
# Call the solver function
source(“e41.R”)
# Solve the full model
sol5 = BLUP(X, y, Z, W, Va, Vpe, Ve, Ai)


Applying Equation (14) instead of Equation (12) requires data transformation. Matrices **Φ**, **A**^*nn*^ and **A**^*np*^ are obtained using function “getPhi” coded in getPhi.R. Solutions of the reduced model are obtained as:


# Call the function to create Phi matrix
source(“getPhi.R”)
# Define phenotyped animals
phe=4:8
# Create Phi and back-solving matrices
mats = getPhi(Ai,phe)
# Solve the reduced model
sol6 = BLUP(X, y, Z=Z[,phe], W, Va,
 Vpe, Ve, Ai=mats$Phi)


Following Equation (6), ${\hat{\text{u}}}_{n}$ are back-solved using the following code:


u_n = solve(mats$Ann, mats$Anp %*%
 -sol6[5:9])


The following command confirms that solutions from the full (Equation 12) and the reduced models (Equation 14) are identical.


source(“check.R”) # Call the function
check(sol5, c(sol6[1:4], u_n,
 sol6[5:14]))


### 3.4. Maternal Trait Animal Models

Data from Example 6.1 of Mrode (2005) was used (Supplementary Table 4). This example includes maternal genetic and maternal permanent environment effects. The data can be produced using e61data.R in the data repository. The equation system is coded in function “BLUP” in e61.R. The following code produces the data and solutions.


# Create data
source(“e61data.R”)
# Call the solver function
source(“e61.R”)
# Solve the full model
sol7 = BLUP(X, y, Z, W, S, G, Vmpe,
 Ve, Ai)


Applying the reduced model requires data transformation. Matrices **Φ**, **A**^*nn*^, and **A**^*np*^ are obtained using function “getPhi” coded in getPhi.R. Solutions of the reduced model are obtained as:


# Call the function to create Phi matrix
source(“getPhi.R”)
# Define phenotyped animals
phe=5:14
# Define dams
dams = c(2,5:7)
# Find being phenotyped or dam
pheORdam = sort(unique(c(phe, dams)))
# Create Phi and back-solving matrices
mats = getPhi(Ai,pheORdam)
# Solve the reduced model
sol8 = BLUP(X, y, Z=Z[,pheORdam],
  W=W[,pheORdam],
  S, G, Vmpe, Ve, Ai=mats$Phi)


Following Equation (21), ${\hat{\text{u}}}_{n}$ and ${\hat{\text{u}}}_{n}$ are back-solved using the following code:


Gi = solve(G)
sol_n = solve(kronecker(Gi, mats$Ann),
       kronecker(Gi, mats$Anp) %*%
       -sol8[5:26])
# Separate direct and maternal genetic
 back-solved solutions
u_n = sol_n[1:3]
m_n = sol_n[-3:-1]


The following command confirms that solutions from the full and the reduced models are identical.


source(“check.R”) # Call the function
check(sol7, c(sol8[1:4], u_n[1],
sol8[5], u_n[2:3],
     sol8[6:15], m_n[1], sol8[16],
     m_n[2:3], sol8[17:30]))


### 3.5. Animal Models With Genetic Groups

Data from Example 3.4 of Mrode (2005) was used (Supplementary Table 5). The data can be produced using e34data.R in the data repository. Equation (25) is coded in function “BLUP” in e34.R. The following code produces the data and solutions.


# Create data
source(“e34data.R”)
# Call the solver function
source(“e34.R”)
# Solve the full model
sol9 = BLUP(X, y, Z, lambda, Ai, Q)


Applying Equation (27) instead of Equation (25) requires data transformation. Matrices **Φ**, **A**^*nn*^, and **A**^*np*^ are obtained using function “getPhi” coded in getPhi.R. Solutions of the reduced model are obtained using function “BLUP” coded in e31.R, and the following code.


# Call the function to create Phi matrix
source(“getPhi.R”)
# Call the solver function for the
 reduced model
source(“e31.R”)
# Define phenotyped animals
phe=4:8
# Create Z matrix for the reduced model
Z = Z[,phe]
Z = cbind(Z, matrix(0,nrow(Z),ncol(Q)))
# Make extended A^-1 including genetic
groups
tmp = cbind(diag(nrow(Q)), -Q)
Ai2 = crossprod(tmp, Ai) %*% tmp
# Create Phi and back-solving matrices
# Reduction to phenotyped animals and
genetic groups
mats = getPhi(Ai=Ai2, c(phe,9))
# Solve the reduced model
 sol10 = BLUP(X, y, Z, lambda,
 Ai=mats$Phi)


Following Equation (29), ${\hat{\text{u}}}_{n}+{\text{Q}}_{n}\hat{\text{g}}$ are back-solved using the following code:


u_n = solve(mats$Ann, mats$Anp %*%
- sol10[-2:-1])


The following command confirms that solutions from the full (Equation 25) and the reduced models (Equation 27) are identical.


source(“check.R”) # Call the function
check(sol9, c(sol10[1:2], u_n,
 sol10[3:8]))


### 3.6. Animal Models With a Genomic Relationship Matrix (GBLUP)

Data from Example 3.1 of Mrode (2005) was used (Supplementary Table 1). The data can be produced using e31data.R in the data repository. Rather than using the **A**^−1^ matrix produced by e31data.R (data repository), a **G**^−1^ matrix was used, created by makeGi.R (data repository). The following code creates the data and solutions. The solutions are obtained using the function “BLUP” in e31.R.


# Create data
source(“e31data.R”)
# Call the solver function
source(“e31.R”)
# Make a G^-1 matrix
source(“makeGi.R”)
# Solve the full model
sol11 = BLUP(X, y, Z, lambda, Ai=Gi)


Applying Equation (31) instead of Equation (30) requires data transformation. Solutions of the reduced model are obtained as:


# Define phenotyped animals
phe=4:8
# Define non-phenotyped animals
nonphe = which(! 1:nrow(ped) %in% phe)
# Make G for phenotyped animals
(reduced model)
G = tcrossprod(M[phe,]-2*P[phe,])/
(2*sum(p*(1-p))) +
   diag(length(phe))/10
# Invert G
Gi2 = solve(G)
# Solve the reduced model
sol12 = BLUP(X, y, Z=Z[,phe], lambda,
Ai=Gi2)


Following Equation (6) (substituting **A** to **G**), ${\hat{\text{u}}}_{n}$ are back-solved using the following code:


u_n = solve(Gi[nonphe,nonphe],
Gi[nonphe,phe] %*%  -sol12[-2:-1])


The following command confirms that solutions from the full (Equation 30) and the reduced (Equation 31) models are identical.


source(“check.R”) # Call the function
check(sol11, c(sol12[1:2], u_n,
sol12[3:7]))


### 3.7. Animal Models With an Augmented Pedigree-Genomic Relationship Matrix (ssGBLUP)

Data from Example 3.1 of Mrode (2005) was used (Supplementary Table 1). The data can be produced using e31data.R in the data repository. It was considered that animals 3, 7, and 8 are genotyped and a 3 × 3 arbitrary $\left({\text{G}}^{-1}-{\text{A}}_{22}^{-1}\right)$ matrix was considered for genotyped animals. An **H**^−1^ matrix was created (Equation 32) to be used instead of **A**^−1^. The following code creates the data and solutions. The solutions are obtained using the function “BLUP” in e31.R.


# Create data
source(“e31data.R”)
# Call the solver function
source(“e31.R”)
# Define genotyped animals
geno = c(3,7,8)
# Make an arbitrary G^-1 - A_22^-1
GimA22i = matrix(c(50,2,-5,2,40,6,-5,6,
60)/100, nrow=3)
# Create H^-1
Hi = Ai
Hi[geno, geno] = Ai[geno, geno]
+ GimA22i
# Solve the full model
sol13 = BLUP(X, y, Z, lambda, Ai=Hi)


Applying Equation (34) instead of Equation (33) requires data transformation. Solutions of the reduced model are obtained as:


# Define phenotyped animals
phe=4:8
# Define phenotyped or genotyped
animals
pheORgeno = sort(union(phe, geno))
# Define non-phenotyped and
non-genotyped animals
nonpheANDnongeno = setdiff(1:nrow(Ai),
pheORgeno)
# Create H^55
H55 = Hi[pheORgeno, pheORgeno]
# Create A^44
A44 = Ai[nonpheANDnongeno,
nonpheANDnongeno]
# Create A^45
A45 = Ai[nonpheANDnongeno, pheORgeno]
# Create Theta matrix (Eq. 34)
Theta = H55 - crossprod(A45,solve(A44))
%*% A45
# Solve the reduced model
sol14 = BLUP(X, y, Z=Z[,pheORgeno],
lambda, Ai=Theta)


Following Equation (35), solutions for non-phenotyped and non-genotyped animals are back-solved using the following code:


u_n = solve(A44, A45 %*% -sol14[-2:-1])


The following command confirms that solutions from the full (Equation 33) and the reduced (Equation 34) models are identical.


source(“check.R”) # Call the function
check(sol13, c(sol14[1:2], u_n,
sol14[3:8]))


## 4. Discussion

Genetic evaluation centers around the world deal with tight deadlines for delivering breeding value predictions of animals to the industry. The amount of data to be dealt with usually contains millions of animals and that can make restraints around computational time and resources. Often, there are many historical or ancestral animals as well as inactive (culled) breeding animals, for which breeding values are not routinely needed. Computational demand can be reduced by applying reduced animal models that are equivalent to the full animal models.

Several forms of animal model were reduced to comprise equations for breeding values only for phenotyped animals, and extension to other forms of animal model can be done following the same logic. The efficiency of these models depends on several factors, such as the proportion of phenotyped animals in the population being evaluated, sparseness of **Φ**, and the number of effects in common between the full and the reduced model. There is also a trade-off between the number of phenotyped and non-phenotyped animals. The smaller the proportion of phenotyped animals the smaller the size of MME, but the greater the computational cost of calculating **Φ** as a result of a larger **A**^*nn*^. The sparsity of **Φ**, which itself depends on the sparsity of **A**^*nn*^ [the sparser the **A**^*nn*^, the sparser the (**A**^*nn*^)^−1^] can heavily reduce the efficiency of the reduced animal model. Depending on the size of **Φ**, it might have more non-zero elements than the **A**^−1^ matrix. This might erode any computational benefit from the reduced model compared to the full model. We suggest two solutions for this problem. One is discarding non-phenotyped animals that have little or no relatedness with phenotyped progeny (i.e., a few generations distant from any phenotyped descendant), and the other is re-ordering **A**^*nn*^ to obtain two diagonal blocks, one very sparse (**A**^*ss*^) and the other (**A**^*dd*^) relatively dense $\left(\begin{array}{c} {\text{A}}^{nn}=\left[\begin{array}{cc} {\text{A}}^{ss} & {\text{A}}^{sd}\\ {\text{A}}^{ds} & {\text{A}}^{dd} \end{array}\right] \end{array}\right).$ Then, **A**^*dd*^, **A**^*sd*^, and **A**^*ds*^ are appended to **A**^*pp*^, **A**^*np*^, and **A**^*pn*^. This also requires appending rows of 0 to **X** and columns of 0 to **Z** (corresponding to the appended non-phenotyped animals). A pedigree-only-based back-solving (such as for BLUP and for ssGBLUP dividing animals to “phenotyped or genotyped” and “non-phenotyped and non-genotyped”) can be done efficiently for millions of animals.

Solutions for non-phenotyped animals can be back-solved after the solutions for phenotyped animals are obtained. Generally, back-solving is more efficient and faster than solving the mixed model equations, and that is due to the known structure of **A**^−1^. Usually, breeding values of only a small proportion of non-phenotyped animals are of interest (e.g., active non-phenotyped breeding animals). Consequently, the back-solving procedure can be omitted by explicitly including those animals together with phenotyped animals in the reduced animal model.

The same reduction technique is applicable to BLUP using the genomic relationship matrix (GBLUP). That would reduce the computational demand to form and invert **G** by limiting it to phenotyped animals only, and the computational demand to solve a smaller set of mixed model equations. Note that the computational cost of inverting **G** is cubic to its dimension unless it is not full rank because it was formed with fewer markers than the number of genotyped animals, where an equivalent model (Fernando et al., 2016) can be used or it uses a reduced rank approximation, such as the algorithms introduced by Misztal (2016) and Mäntysaari et al. (2017). Though, back-solving is possible, it is discouraged as it involves the availability of the blocks of **G**^−1^ among non-phenotyped animals and between non-phenotyped and phenotyped animals. Because most of the computational complexity of GBLUP is due to forming and inverting **G**, it is recommended to form and invert **G** for phenotyped animals together with non-phenotyped animals of interest. However, it is assumed that either the allele frequencies in the whole population are known or allele frequencies in the individuals present in the reduced model are not different from the allele frequencies in the whole population. Also, as it was mentioned earlier, another way of back-solving for non-phenotyped animals is through back-solving of marker effects. There are other forms of reduced GBLUP proposed by other studies (Cantet and Smith, 1991; Hoeschele, 1993; Arendonk et al., 1994; Saito and Iwaisaki, 1996, 1997). Comparing the efficiency of these methods and possibly combining them with the reduced GBLUP presented in this study are yet to be investigated. The computational efficiency of the reduced GBLUP presented in this study heavily depends on the proportion of animals not included in the reduced model (genotyped and non-phenotyped animals whose evaluation is not of interest).

There are two possibilities for ssGBLUP; dividing animals to phenotyped and non-phenotyped animals or dividing animals to “phenotyped or genotyped” and “non-phenotyped and non-genotyped” groups. The latter is preferred as it makes back-solving independent from **H**^22^ (block of **H**^−1^ corresponding to genotyped animals) and dependent to blocks of **A**^−1^ between the two groups of animals and among “non-phenotyped and non-genotyped” animals. Blocks of **A**^−1^ are much easier to calculate and keep in the memory, because of its sparsity.

Variance components can be estimated using reduced animal models (e.g., Henderson, 1986; Besbes et al., 1992; White et al., 2006; Guy et al., 2009). Restricted maximum likelihood (REML, Patterson and Thompson, 1971) has been a popular method for variance components estimation. Generalized inverses of coefficient matrices of mixed model equations are required under both the full and the reduced animal models (Henderson, 1986). Reduced animal models have considerably smaller order coefficient matrix and consequently less computational complexity of variance components estimation. Henderson (1986) estimated variances for the reduced model based on the method of Quaas and Pollak (1980). Parents with phenotyped progeny are evaluated in this reduced model. To account for Mendelian sampling and for the genetic contribution of unknown parents, there is an additional component (compared to the full model) to the variance of residuals (Henderson, 1986). The introduced reduction method in this study evaluates all the phenotyped animals and no extra assumption or component is required to hold the assumption on *var*(**y**). The matrices used in the reduced model do not make any deviation from the assumptions on variance components. Also, creating the **Z** matrix for the reduced model in this study is trivial (if the vector of phenotypes is ordered by the order of animals in the pedigree, **Z** is an identity matrix with the order of the number of phenotypes; for a repeatability model, it is still an incidence matrix with coefficients 0 and 1, relating phenotypes to animals). Creating the **Z** matrix for the reduced model of Quaas and Pollak (1980) requires pedigree information.

The presented reduced animal models can further be reduced. This can be achieved by a “2-fold” model reduction, which involves reducing the model to equations for phenotyped animals followed by reducing the model to parents of phenotyped animals (Quaas and Pollak, 1980). Such 2-fold reduced animal model contains equations corresponding to phenotyped parents of phenotyped animals. A 2-fold reduced animal model also requires a 2-fold back-solving procedure, in which the solutions of non-phenotyped parents are back-solved from the solutions of phenotyped parents, and then the solutions of non-parents (and parents of non-phenotyped progeny) are back-solved from the solutions of parents. This 2-fold back-solving can be omitted or reduced to a 1-fold back-solving by putting together a group of animals of interest to phenotyped parents with phenotyped progeny. Further research is required on this subject.

Finally, depending on the size of the data and the number of traits and effects in the model, the equation system of the reduced animal model may become small enough that direct solving of the mixed model equations might become faster and more efficient than iterative procedures for solving mixed model equations (Schaeffer and Kennedy, 1986; Berger et al., 1989; Strandén and Lidauer, 1999).

## Data Availability Statement

The R scripts to produce the data and perform the analyses for this study can be found in the figshare repository [https://doi.org/10.6084/m9.figshare.13369607].

## Author Contributions

DG initiated the idea of the study. MN extended the idea to other forms of animal model, drafted the manuscript, coded the examples, and validated the results. Both DG and MN took active part in the preparation and revision of the manuscript.

## Conflict of Interest

MN is employed by the company Livestock Improvement Corporation, Hamilton, New Zealand. The remaining author declares that the research was conducted in the absence of any commercial or financial relationships that could be construed as a potential conflict of interest.
