# Supplementary material for: Reduced Animal Models Fitting Only Equations for Phenotyped Animals
Source: Front Genet. 2021 Mar 22;12:637626. doi: 10.3389/fgene.2021.637626 (PMC8019830; doi:10.3389/fgene.2021.637626)
Supplement: Supplementary file 1 [file Table_1.PDF]

Supplementary Material

Table S1. Pre-weaning gain (WWG) for a group of beef calves (Mrode, 2005)*.

Calf	Sex	Sire	Dam	WWG
4	Male	1	–	4.5
5	Female	3	2	2.9
6	Female	1	2	3.9
7	Male	4	5	3.5
8	Male	3	6	5.0

* Direct additive genetic and residual variances equal to 20 and 40, respectively.

Table S2. Pre-weaning gain (WWG) and post-weaning gain (PWG) for a group of beef calves (Mrode, 2005)*.

Calf	Sex	Sire	Dam	WWG	PWG
4	Male	1	–	4.5	–
5	Female	3	2	2.9	5.0
6	Female	1	2	3.9	6.8
7	Male	4	5	3.5	6.0
8	Male	3	6	5.0	7.5
9	Female	7	8	4.0	–

* Genetic and residual variance-covariances equal to $\begin{bmatrix} 20 & 18 \\ 18 & 40 \end{bmatrix}$ and $\begin{bmatrix} 40 & 11 \\ 11 & 30 \end{bmatrix}$, respectively, with the first trait being WWG.

Table S3. Fat yield (FY) for a group of cows in a single dairy herd (Mrode, 2005)*.

Cow	Sire	Dam	Parity	Season	FY
4	1	2	1	1	201
4	1	2	2	3	280
5	3	2	1	1	150
5	3	2	2	4	200
6	1	5	1	2	160
6	1	5	2	3	190
7	3	4	1	1	180
7	3	4	2	3	250
8	1	7	1	2	285
8	1	7	2	4	300

* Direct additive genetic, permanent environment and residual variances equal to 20, 12 and 28, respectively.

Table S4. Birth weight (BW) for a group of beef calves (Mrode, 2005)*.

Calf	Sire	Dam	Herd	Sex	BW
5	1	2	1	Male	35
6	3	2	1	Female	20
7	4	6	1	Female	25
8	3	5	1	Male	40
9	1	6	2	Male	42
10	3	2	2	Female	22
11	3	7	2	Female	35
12	8	7	3	Female	34
13	9	2	3	Male	20
14	3	6	3	Female	40

* Direct additive genetic, maternal genetic, permanent environment and residual variances equal to 150, 90, 40 and 350, respectively, and the covariance between direct additive and maternal genetic effects equal to 40.

Table S5. Pre-weaning gain (WWG) for a group of beef calves with two genetic groups (G1, G2) in the pedigree (Mrode, 2005)*.

Calf	Sire	Dam	Sex	WWG
1	G1	G2	Male	–
2	G1	G2	Female	–
3	G1	G2	Male	–
4	1	G2	Male	4.5
5	3	2	Female	2.9
6	1	2	Female	3.9
7	4	5	Male	3.5
8	3	6	Male	5.0

* Direct additive genetic and residual variances equal to 20 and 40, respectively.
